# Beyond standard model calculations with Sherpa

**DOI:** 10.1140/epjc/s10052-015-3338-4

**Published:** 2015-03-24

**Authors:** Stefan Höche, Silvan Kuttimalai, Steffen Schumann, Frank Siegert

**Affiliations:** 1 SLAC National Accelerator Laboratory, Menlo Park, CA 94025 USA; 2 Institute for Particle Physics Phenomenology, Durham University, Durham, DH1 3LE UK; 3 II. Physikalisches Institut, Universität Göttingen, Friedrich-Hund-Platz 1, 37077 Göttingen, Germany; 4 Institut für Kern- und Teilchenphysik, TU Dresden, 01062 Dresden, Germany

## Abstract

We present a fully automated framework as part of the Sherpa event generator for the computation of tree-level cross sections in Beyond Standard Model scenarios, making use of model information given in the Universal FeynRules Output format. Elementary vertices are implemented into C++ code automatically and provided to the matrix-element generator Comix at runtime. Widths and branching ratios for unstable particles are computed from the same building blocks. The corresponding decays are simulated with spin correlations. Parton showers, QED radiation and hadronization are added by Sherpa, providing a full simulation of arbitrary BSM processes at the hadron level.

## Introduction

The quest for new-physics signals in collider data requires their detailed simulation. Comprehensive analyses of measurement sensitivities, exclusion limits or possibly anomalies often consider a variety of Beyond Standard Model (BSM) scenarios. For each hypothesis, production cross sections need to be evaluated, and particle decay widths and branching ratios have to be computed. Realistic simulations further include spin correlations between production and decay. For simulations at the particle level, parton-shower effects and non-perturbative corrections must also be considered.

Given the vast number of new-physics models, the automation of such calculations is mandatory. In fact, in the past years enormous efforts were made not only to automate leading-order calculations, but next-to-leading-order calculations as well. A variety of related tools have been constructed, ranging from Feynman rule generators like FeynRules [1, 2] over spectrum-generator generators like Sarah [3, 4] to matrix-element generators like MadGraph [5], MadGolem [6, 7], MadLoop [8, 9], Whizard [10] and Amegic [11] and particle-level event generators [12], such as Herwig [13, 14], Pythia [15, 16] and Sherpa [17, 18]. Each of them deals with particular aspects of the simulation. Specific protocols have been developed to guarantee consistent parameter and event passing between the various tools [19–21].

In this paper we present the status and new developments regarding the simulation of new-physics signals with the event generator sherpa  [17, 18]. Former versions of sherpa already supported quite a number of new-physics models. They were either built in as for example the MSSM [22], the ADD model [23] and several others [24–26], or invoked through a dedicated interface to FeynRules [27]. This interface was limited to vertices with color- and Lorentz-structures supported by the matrix-element generator Amegic [11]. In the work presented here we lift these restrictions by extending the capabilities of sherpa ’s second built-in matrix-element generator Comix [28] to account for almost arbitrary BSM scenarios. We generalize the recursive amplitude generation formalism to arbitrary $n$-point vertices, and we automate the implementation of Lorentz calculators based on the model representation in the Universal FeynRules Output (UFO) [29]. Part of our new generator is thus equivalent to Aloha [30]. At present we constrain ourselves to particles of spin-0, spin-1/2 and spin-1. A generalization to spin-3/2 and spin-2 states is straight-forward and foreseen for the near future. Similarly, we restrict ourselves to color structures involving singlets, (anti-)triplets, and octets. (Anti-)sextet representations will be included in the near future. We also discuss the implementation of an algorithm to preserve spin correlations between factorized production and decay processes [31].

This paper is organized as follows. In Sect. 2 we discuss the techniques used for amplitude generation focusing on the newly developed methods for the automatic implementation of Lorentz structures. We also present the results of an extensive validation. In Sect. 3 we introduce and discuss our treatment of particle decays, including spin-correlation effects. After a discussion of other event generation aspects in Sect. 4 the conclusions and an outlook are given in Sect. 5.

## Cross-section calculations at tree-level

This section briefly describes the algorithms implemented in the matrix-element generator Comix to compute tree-level amplitudes. Identical methods are used to obtain tree-level like objects for next-to-leading order calculations, i.e. the color-correlated Born amplitudes entering dipole-subtraction terms in the Catani–Seymour method [32, 33] or the FKS method [34]. The implementation of dipole-subtraction in Comix will be described elsewhere [35].

A recursive algorithm for the computation of color-ordered multi-parton amplitudes was proposed long ago [36, 37]. Its extension to colorful amplitudes [38] leads to a recursion that resembles the Dyson–Schwinger equations [39–41]. In this publication we extend the implementation of the algorithm in the matrix-element generator Comix [28] such that it can handle $n$-point vertices at tree level, where $n$ is – in principle – unbounded. The automatic implementation of related Lorentz structures is described in Sect. 2.2.

**Fig. 1:**
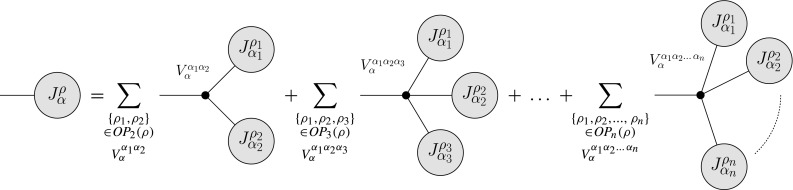
Sketch of the Berends–Giele type recursive relation as implemented in Comix. The current $J^\rho _\alpha$ is computed as a sum over sub-currents joined by elementary vertices. This formulation is inherently recursive. The sums on the *right-hand side* extend over all ordered partitions of the set of particles, $\rho$, on the *left hand side*. The multi-indices $\alpha$ denote both Lorentz and color indices of the currents. Displayed are vertices with up to $n+1$ external particles

Schematically the algorithm to compute tree-level amplitudes based on the Berends–Giele type recursive relations is depicted in Fig. 1. Consider an unordered $N$-particle current, $J^\rho _\alpha$, where $\rho$ denotes the set of $N$ particles, and $\alpha$ is a multi-index that labels both Lorentz and color indices of the current. This current is computed from all Feynman graphs having as external particles the on-shell particles in the set $\rho$, and the (potentially off-shell) particle described by $J^\rho _\alpha$. Special currents are given by the external-particle currents. They correspond to the helicity Eigenvectors of wave functions for the external particles, as described in [28]. Assuming that up to $n+1$-point vertices exist, off-shell currents can be computed as

2.1 $$\begin{aligned}&J^\rho _\alpha \;=\;P^{\,\rho }_{\alpha }\; \sum _{m=2}^n\; \sum \limits _{\begin{array}{c} \{\rho _1,\ldots ,\,\rho _m\}\\ \in \, O\!P_m(\rho ) \end{array}}\; \sum \limits _{V_{\alpha }^{\,\alpha _1\ldots \,\alpha _m}}\;\mathcal {S}(\rho _1,\ldots ,\,\rho _m)\;\nonumber \\&\times \,V_{\alpha }^{\,\alpha _1\ldots \,\alpha _m}\;J^{\rho _1}_{\alpha _1}\,\ldots \,J^{\rho _m}_{\alpha _m}. \end{aligned}$$

Here $P^\rho _\alpha$ denotes a propagator term depending on the particle type $\alpha$ and the set of particles $\rho$. The sum over $V$ extends over all elementary vertices of the theory that have as external states the particles described by the currents $J^{\rho _1}_{\alpha _1}\ldots J^{\rho _m}_{\alpha _m}$. For some assignment of currents no such vertex may exist. The final sum extends over all ordered partitions of the set of indices in $\rho$. $\mathcal {S}$ is the symmetry factor associated with the decomposition of $\rho$ into subsets, see [28].

An $N$-particle scattering amplitude is given in terms of the above current as

2.2 $$\begin{aligned} \mathcal {A}(1,\ldots ,N)\,=\;J^{\{N\}}_\alpha \, \frac{1}{P^{\{1,\ldots ,N-1\}}_\alpha }\, J^{\,\{1,\ldots ,N-1\}}_\alpha . \end{aligned}$$

Note that the amputation of the final propagator term is schematic. In practice, one does not multiply with this term in the first place.

In order to implement Eqs. (2.1) and (2.2) we employ the spinor basis introduced in Ref. [42]. The $\gamma$-matrices are taken in the Weyl representation, which has the advantage that massless spinors are described by only two nonzero components. Polarization vectors for external vector bosons are constructed according to Ref. [43].

Majorana fermions are treated in the formalism of [44, 45]. Their external wave functions can be constructed either as if they represent fermions, or as if they represent anti-fermions. This is left optional in Comix, and it can be used to check the consistency of the calculation.

Comix allows to specify coupling orders for the calculation. This permits, for example, to compute only strongly interacting parts of $pp\rightarrow jj$ amplitudes, or exclusively electroweak contributions. In the UFO format, not only the QCD and electroweak order of a coupling can be specified. Instead, arbitrary orders can be defined and the coupling constants are classified accordingly. This feature is fully supported and by default no restrictions with respect to coupling orders are applied. If instead the user specifies a coupling constraint, Comix applies this constraint at the amplitude-squared level. It is therefore also possible to compute pure interference terms. While these terms are not observable in practice, computing them is often instructive to study directly the difference between coherent and incoherent sums of signal and background contributions.

### Treatment of color

Comix samples external colors and performs the color algebra in the color-flow decomposition at the vertex level. The color-flow decomposition, formally introduced in [46], was advertised in the context of collider physics in [47]. It was shown to be superior for high-multiplicity QCD calculations in [38].

In the color-flow decomposition, each particle in the adjoint representation is replaced by a bi-fundamental, while keeping track of the active degrees of freedom by applying projection operators. This amounts to cutting adjoint propagators by inserting the identity $\delta ^{ab}=T^a_{ij}T^b_{ji}$ and identifying $i$ and $j$ as the propagator indices. In practice one contracts adjoints with generators at vertices, while inserting projectors of the form $T^a_{ij}T^a_{kl}$ in each propagator.

We have implemented the relevant color structures for the standard model, the MSSM, and a range of BSM theories. This includes the trivial identities, group generators, structure constants as well as simple products of those. Color (anti-)sextets can be accomodated, but our code does not include them at present. The implementation of Standard Model color structures has been detailed in [38]. It is straightforward to implement higher-point functions, and the corresponding objects can be supplied to Comix at runtime using a dynamically linked library. So far we have not automated the generation of color calculators, but there is no obstacle to do so.

### Automatic implementation of Lorentz calculators

**Fig. 2:**
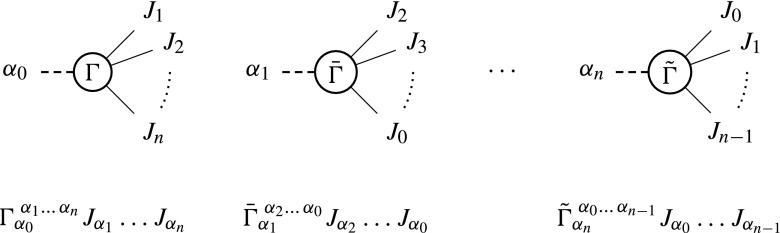
Pictorial representation of the possible rotations of Lorentz calculators

Within the Dyson-Schwinger formalism discussed above, any off-shell current corresponds to a particle and therefore one specific quantum field and its representation of the Lorentz group. Although the implementation in Comix is currently limited to spin-0, spin-1/2, and spin-1 particles (including the spin-2 pseudoparticle described in [38]), our automatic implementation of numerical routines for evaluating the Lorentz structures of vertices is generic. It only requires, that currents be represented by multi-component complex objects and that the recursive relations, Eq. (2.1), are used. For each model, routines must be provided that evaluate expressions of the form $\Gamma _{\alpha _0}^{\;\alpha _1\ldots \,\alpha _n}J_{\alpha _1}\ldots J_{\alpha _n}$, which correspond to the space-time structure of the vertices in Eq. (2.1). Pictorially, one can represent such terms as shown in Fig. 2.

The Universal FeynRules Output (UFO) [29] is a format for exchanging information on interaction vertices in terms of a basic set of color and Lorentz structures and symbolic algebraic operations on those. We have constructed a Python module that implements explicit representations of the Lorentz structures as they are used in Comix and maps them onto the definitions in the UFO. This module is capable of performing all algebraic operations on these building blocks to generate C++ source code to be used by Comix for the corresponding Lorentz calculators.

With the UFO expression for an $n+1$-particle vertex at hand, the Python module sets up external currents $J_{\alpha _1},\ldots ,J_{\alpha _n}$ with symbolic components and then performs the multiplications and implicit sums over indices, leaving only the “outgoing” index, $\alpha _0$, uncontracted. This yields an explicit expression for all components of the current $J_{\alpha _0}$ that is stored in the form of C++ code. Note that this procedure needs to be performed for all cyclic permutations of indices $\{0,\ldots ,n\}$, each one corresponding to a different “outgoing” index. Pictorially, this corresponds to a counter-clockwise rotation of the vertex, as shown in Fig. 2.

As an example, consider the gauge coupling of a vector field $A^\mu$ to a fermion, $\overline{\psi }\gamma _\mu A^\mu \psi$. Taking $\alpha _0$ to be the vector, and $\alpha _1$ and $\alpha _2$ to be the Dirac antiparticle and particle, respectively, the Lorentz calculator schematically depicted in Fig. 2 would correspond to2.3Analogous expressions must be provided for the other two cyclic permutations of the indices $\{0,1,2\}$.

### Implementation of model parameters

The C++ routines generated in this manner are compiled and linked along with the information on the particle content of the model and the model parameters. The dynamic library containing Lorentz calculators and model information is loaded by sherpa at program startup. The entire process is automated to a high level, such that the user needs to run just a single command to make the entire UFO model available for event generation.

The parameters of the model are set to the default values given in the UFO. They can be overwritten at runtime using a file which largely follows the SLHA [19–21]. Note that at this level it is not possible anymore to change parameters which would lead to the appearance of additional vertices in the model, like changing the Yukawa mass of a bottom quark from zero to a nonzero value. The set of model parameters is available throughout the whole sherpa framework, which guarantees the consistent use of couplings and particle masses at all stages of event generation.

### Illustrative examples

In order to validate our new generator we compared numerous results obtained with Comix for a variety of models against Amegic [11] and MadGraph5 [5].

**Fig. 3:**
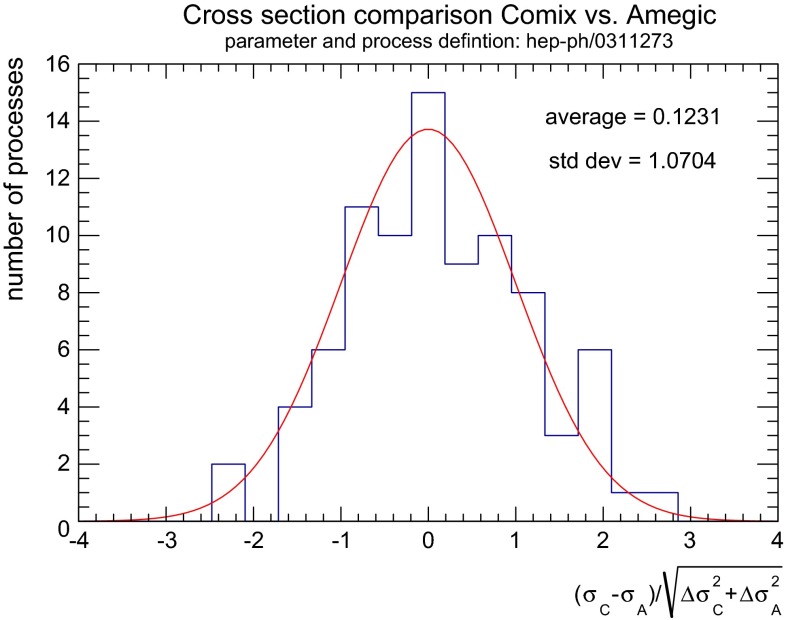
Deviation between results from Amegic and Comix for the 86 $e^+e^-\rightarrow 6f$ processes listed in Ref. [48], using the parameters given ibidem. The *red curve* represents a normal distribution and should be considered the reference

Figure 3 shows the deviation of leading-order cross sections computed both with Amegic and Comix for the 86 $e^+e^-$ to six-fermion processes listed in Ref. [48], where each result is computed to better than $5\,\permille$ Monte-Carlo uncertainty. It can be seen that the deviation between the two generators is of purely statistical nature. This confirms the correct implementation of the Standard Model in the extended version of Comix, and it validates the recursive phase-space generator described in [28].

Table 1 presents a comparison of tree-level matrix elements between Comix and MadGraph5. In all tests we have considered 1000 individual phase-space points per process. For each model we quote only the maximal deviation found when comparing matrix elements from MadGraph5 and Comix. We considered the processes and parameters listed in [27] for the Minimal Universal Extra Dimensions Model. In case of the MSSM, we tested the more comprehensive set of processes considered in [22] and the set of processes considered in [27] for the Standard Model was also supplemented by further $2\rightarrow 2$, $2\rightarrow 3$, and $2\rightarrow 4$ processes.

**Table 1:** Maximal relative deviations between tree-level matrix elements computed with Comix and MadGraph5. For each model we quote the largest observed deviation among all processes, where we tested 1000 random phase-space points per process

Model	Number of processes tested	Max. rel. deviation Comix $\leftrightarrow$ MadGraph5
Standard Model	60	$2.3\times 10^{-10}$
Higgs Effective Field Theory	13	$4.3\times 10^{-13}$
MSSM	401	$1.0\times 10^{-10}$
Minimal Universal Extra Dimensions	51	$2.8 \times 10^{-12}$
Anomalous Quartic Gauge Couplings	16	$5.9\times 10^{-12}$

We have also compared the results from Comix against those from MadGraph5 for two effective theories. The first is based on the Standard Model including couplings of a scalar and a hypothetical pseudoscalar Higgs boson to gluons via a top-quark loop [49–52]. This theory involves up to five-point vertices. In order to test our algorithms in the context of more complicated Lorentz structures and high-multiplicity vertices, we considered anomalous quartic gauge couplings [53–55]. Specifically, we used a model implementing the interaction terms (A7)–(A10), as described in [56]. They give rise to up to eight-particle vertices extending the gauge sector of the Standard Model. We tested $2\rightarrow 2$ as well as $2\rightarrow 4$ processes that are sensitive to complicated Lorentz structures of up to 6-particle vertices which cannot be mapped to Standard-Model like interactions. The number of processes compared and the maximal relative deviation observed are again listed in Table 1. This successful validation proves that effective operators can efficiently be implemented in Comix via FeynRules and UFO.

## Decay simulation including spin correlations

It is often not feasible to simulate new-physics signals at the level of stable final-state particles. The possibility of many intermediate resonances leads to a large number of different final states. Even if matrix-element calculation and phase-space integration for each of those final states are in principle feasible, the management of all possible states within a matrix-element generator becomes computationally challenging and practically useless. It is more convenient to simulate only the production of certain new-physics resonances, and possibly the accompanying hard QCD and/or QED radiation, while treating the cascade decay of heavy unstable new-physics objects in a different manner.

Here we describe a module of the sherpa event generator which implements such a decay cascade. It performs two main tasks which will be described in the next subsections: the construction of the cascade itself, and the preservation of spin correlations which are neglected during the independent calculation of production and decay in the cascade.

### Construction of the decay cascade

To construct a decay cascade one recursively simulates single decay processes until only stable particles are left. For the simulation of each single decay process several ingredients are necessary.

The first step is the choice of a decay channel according to its branching ratio. The basic information for determining possible decay modes of a given unstable particle $P$ are the vertices, $V$, of Eq. (2.1), which contain $P$ among their $n$ external lines. Using these vertices as a starting point, an initial (direct) decay table is built up for potential $P\rightarrow n-1$ decay modes.

Each decay mode can then be revisited to decide whether it is accepted as final or whether it should be replaced by including further iterative decays.^1^ The simplest option for this decision is the mass threshold criterion: if the mass of the outgoing system is larger than the decayer mass, then the direct decay mode is discarded and replaced by all possible combinations where one final-state particle has been replaced by its own decay products. When a decay mode is replaced, only diagrams with the given propagator structure should be included in the matrix elements for the new decay channels. For cases where the threshold criterion is too simple an alternative option is implemented where the decision is triggered by a comparison of the partial widths calculated from the direct vs. the converted decay modes. If more sophisticated threshold behavior is necessary the user of sherpa can implement a dedicated trigger criterion involving e.g. additional phase-space weights. This conversion of decay modes could be iterated. In our implementation we allow for one step, which should be sufficient for most practical applications. Assuming e.g. only 3-point vertices for simplicity this allows for a conversion from $1\rightarrow 2$ modes to $1\rightarrow 3$ modes. Depending on the complexity of the model it can take a few minutes to construct the decay table. Considering for example the MSSM model with the SPS1a benchmark point [57], we find that the construction of the decay table takes 150 seconds using one core of an Intel Xeon E5-2670 CPU at 2.6 GHz and requires 0.7 GB of main memory. To facilitate a quick initialization for the case of more complex models it is possible to write the decay table to disk and read it back in.

For each final decay channel the corresponding matrix element is constructed using the building blocks described in Sect. 2. This implies that the full BSM capabilities stemming from the UFO implementation are available also in the decay module. We consider tree-level amplitudes only, using the exact same model parameters as for the hard-scattering process, cf. Sect. 2.3. Integrating a decay matrix element over phase space one obtains the partial width of that channel and correspondingly its selection probability in the decay table.

These matrix elements are also used to go beyond an isotropic distribution of the decay kinematics. For simple two-body decays, the phase space is generated using the Rambo algorithm [58]. For decays to three and more particles we employ importance-sampling based on information about propagators [59]. If applicable several channels are combined into a multi-channel integrator [60]. The matrix elements are then used in an unweighting step to provide the final decay kinematics.

The full amplitude-level information including the helicity dependence is also made available to allow for the implementation of spin correlations, as will be described in the following section.

As an additional option to improve the modeling of decay cascades we implement a crude estimation of off-shell effects by adjusting the decay kinematics a posteriori to yield a Breit–Wigner distribution of the decayer momentum. This is at the present based on a constant-width approach and can in the future be improved with dedicated line-shape modeling in selected cases.

### Spin-correlation algorithm

The factorization into production and decay matrix elements is based on the replacement of intermediate particle propagators by a helicity sum, using completeness relations. For example, a full matrix element containing a massive vector-boson propagator can be factorized as:

3.1 $$\begin{aligned} \mathcal {M} \;\sim \; j_1^\mu \,\left[ \,g_{\mu \nu }-\frac{p_\mu p_\nu }{p^2}\,\right] \,j_2^\nu \;=\; \sum \limits _{\lambda } \underbrace{j_1^\mu \varepsilon _\mu ^*(\lambda )}_{\mathcal {M}_\text {prod}(\lambda )} \; \underbrace{\varepsilon _{\nu }(\lambda ) j_2^\nu }_{\mathcal {M}_\text {dec}(\lambda )}. \end{aligned}$$

Similar equations hold for all particle types. If spin correlations were neglected, the common sum over helicities $\lambda$ would be replaced by individual sums for production and decay. While for some applications this is a reasonable approximation, in other cases it will lead to a significant mis-modeling, e.g. of angular correlations between decay products. To remedy this situation we employ a spin-correlation algorithm originally introduced for QCD parton showers [61–63] and generalized to arbitrary decay cascades in [31].

In this algorithm, the helicity summation or averaging in a matrix element is replaced at each step by a contraction with the spin-density matrices $\rho _{\lambda \lambda '}$ of the incoming particles and the decay matrices $D_{\lambda \lambda '}$ of unstable outgoing particles:

3.2 $$\begin{aligned} \frac{\mathrm{d}\Gamma (0\rightarrow 1\dots n)}{\mathrm{d}\Omega } = \rho _{\lambda _0\lambda '_0}\; \mathcal {M}_{\lambda _0;\lambda _1,\dots \lambda _n}\mathcal {M}^*_{\lambda '_0;\lambda '_1,\dots \lambda '_n}\; \prod _{i=1,n} D^i_{\lambda _i\lambda '_i}.\nonumber \\ \end{aligned}$$

These are not fully known at all stages of the decay cascade though, and [31] describes the algorithm with which they can be continually updated and implemented as they become available.

We obtain the full helicity structure of the amplitudes $\mathcal {M}_{\lambda _0;\lambda _1,\dots \lambda _n}$ from our decay matrix-element generator described in Sect. 3.1. We use the same building blocks and gauge conventions in the production and decay matrix elements, therefore the algorithm will directly recover the spin correlations in the decay cascade.

To demonstrate these features, we are presenting one example in the Standard Model, namely top-quark pair production, and one in the MSSM, namely the production of a squark pair with subsequent decay cascades.

#### Top-quark pair production in the SM

For our simulation of top-quark production at the LHC we consider exclusively the decay $t\rightarrow Wb$, while the resulting $W$-boson pair decays into an electron and muon final state according to $W^+ \rightarrow e^+ \nu _e$ and $W^-\rightarrow \mu ^- \bar{\nu }_\mu$. We present results for three different approaches to simulate this final state:

- **Full ME** The full $pp\rightarrow e^+ \nu _e \mu ^- \bar{\nu }_\mu b \bar{b}$ final state is simulated in the Comix matrix-element generator, with a restriction to doubly-resonant diagrams and onshell intermediate top quarks and $W$ bosons. This automatically includes all helicity correlations by construction and is thus used as a reference.
- **Correlated decays** Only the $pp\rightarrow t\bar{t}$ process is generated as hard scattering with the Comix generator. The decays are simulated in a factorized manner and spin correlations are implemented as described above.
- **Uncorrelated decays** As above, but without implementing spin-correlations.

**Fig. 4:**
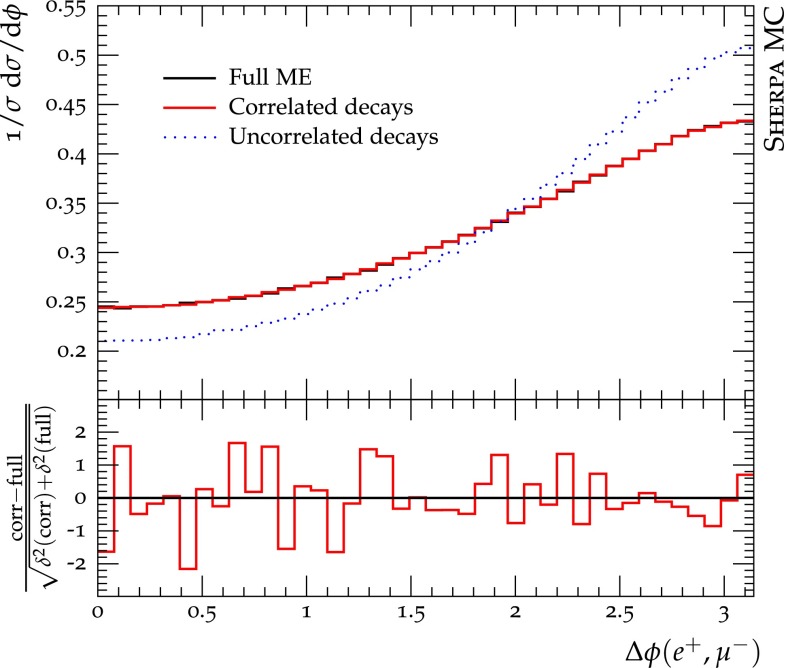
Spin-correlation effects in top-quark pair production in the SM. The three simulation setups are described in the text. The ratio plot displays the relative difference in terms of the standard deviation and allows to judge the statistical compatibility between the full ME and correlated decay simulation

In Fig. 4 we present the comparison of the three different approaches for the azimuthal separation of the muon and positron. It can already be seen in this simple example that the simulation without spin correlations fails to reproduce the correct shape of spin-sensitive observables. With the implementation of the correlation algorithm the decay simulation becomes consistent with the full (resonant) matrix element.

#### Squark pair production in the MSSM

To study spin correlations in a longer decay chain we now turn to the example of squark pair production in the MSSM. We consider scalar up-quark production at the LHC, i.e. $pp \rightarrow \tilde{u}\tilde{u}^*$, with subsequent decays featuring intermediate neutralino and chargino states, i.e.

- $$\tilde{u} \rightarrow d\,\chi _1^+ \left[ \rightarrow \chi _1^0 \, W^+ \left[ \rightarrow \mu ^+ \, \nu _\mu \right] \right] $$ ,
- $$\tilde{u}^* \rightarrow \bar{u}\,\chi _2^0 \left[ \rightarrow e^+\,\tilde{e}^-_R \left[ \rightarrow e^-\,\chi _1^0 \right] \right] $$ .

The full final state studied thus reads $pp \rightarrow \tilde{u}\tilde{u}^* \rightarrow d\,\chi _1^0\,\mu ^+\,\nu _\mu \;\bar{u}\,e^+\,e^-\,\chi _1^0$. The relevant correlations are in particular the ones along the neutralino and chargino propagators. We again compare the three different types of simulation described in Sect. 3.2.1. For our rather technical comparison we consider the MSSM spectrum for the SPS1a benchmark point [57].

**Fig. 5:**
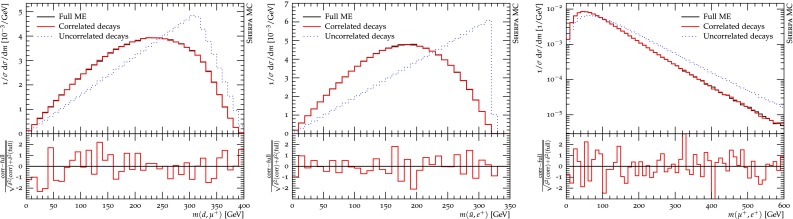
Spin-correlation effects in the decay cascade following squark pair production in the MSSM. The three simulation setups are described in the main text. The ratio plot displays the relative difference in terms of the standard deviation and allows to judge the statistical compatibility between the full ME and correlated decay simulation

Figure 5 shows three different observables which are sensitive to spin correlations. The left panel displays the invariant mass of muon and down quark, an observable which tests correlations within the $\tilde{u}$ decay chain. The middle figure shows a similar observable for the $\tilde{u}^*$ decay chain. The invariant mass of muon and electron displayed in the right panel demonstrates the impact of spin correlations in each decay chain on an observable that correlates both.

## Other aspects of event generation

Any simulation of new physics at the parton level must be embedded into the full event generation at particle level in order to provide realistic final-state information that is suitable for passing to a detector simulation and experimental analysis.

The combination of hard matrix elements with parton showers has been described in some detail in [64, 65]. In the context of new-physics simulations it is often necessary to amend the merging of matrix elements and parton showers with the requirement that no new resonances be present at higher multiplicity. This can be achieved in sherpa using a diagram filter, corresponding to the diagram-removal method described in [66, 67].

Our simulation also includes parton-shower effects in the decay cascade. To account for the fact that in such a case both external and intermediate particles can radiate QCD quanta we use truncated showers [64, 68] on the intermediate states. The input configuration for such a shower simulation is a branching history starting with the hard $2\rightarrow n$ process with resummation scale $\mu _Q$. For each decay process new “layers” are added to this configuration, encoding the $2\rightarrow n+1$, $2\rightarrow n+2$, ...final states, each with a corresponding new resummation scale for the parton shower, that is given by the mass of the particle setting the kink in the color flow. In the case of $t\rightarrow Wb$ decays, this would be the $W$-boson mass, for example.

Note that we implement parton showers in production only, not in decay. This means that for each decaying particle the parton shower is performed from the resummation scale in its production process to the particle width. The same particle does not radiate again during its own decay, which would in principle be required [69]. The mismatch resulting from this approximation is typically small, and we plan to include the missing effects in the near future. Earlier versions of Sherpa, which were based on a different parton shower [70], did indeed include the corresponding algorithm [18, 71].

In addition to the QCD parton shower, sherpa also simulates QED emissions using the YFS algorithm, as detailed in [72]. This is done before the parton shower is implemented.

Ultimately, sherpa invokes a cluster hadronization model [73] to account for the fragmentation of partons into hadrons. However, our hadronization routines can only handle colored Standard-Model partons so far. Other long-lived or even stable colored particles that hadronize, as for example present in various supersymmetric models [74, 75], cannot be dealt with at present.

## Summary and outlook

In this publication we described the methods used to implement arbitrary new-physics models into the event generator sherpa. We provide an automatic generator for Lorentz calculators, which allows to implement interaction vertices which are not present in either the Standard Model or simple extensions thereof. We also extend the matrix-element generator Comix, such that arbitrary higher-point functions can be used for amplitude generation. The new generator supports the Universal FeynRules Output, which is provided by programs like FeynRules and Sarah.

The new and extended version of Comix described here, together with the newly constructed decay module of sherpa, allows to compute the production and decay of new-physics particles, with spin correlations and off-shell effects in the decay taken into account. The simulation is embedded in the larger event generation framework of sherpa to also include QCD radiative corrections by means of the parton shower, QED radiative corrections by means of the YFS approach, and non-perturbative effects through cluster hadronization and hadron decays. Overall, we provide a complete framework to address many new-physics simulations in a fully automated way. Currently our implementation is restricted to spin-0, spin-1/2 and spin-1 particles, but the addition of higher-spin states is foreseen for the near future.

## Footnotes

1 This implies that the decay tables are initialized in the order of the unstable particle masses.
